# Etymologia: *Yersinia*

**DOI:** 10.3201/eid1603.ET1603

**Published:** 2010-03

**Keywords:** etymology, Etymologia, Yersinia

## [yər-sin¢-e-ə]

This genus of gram-negative bacteria was named after bacteriologist Alexandre-Émile-John Yersin (1863–1943). Born in Switzerland, he studied medicine in Paris and began a successful early career in the laboratory. He worked on rabies with Pierre Roux and on the tubercle bacillus under Robert Koch in Germany. He later worked at the Institut Pasteur on the toxic properties of the diphtheria bacillus and eventually signed on as a doctor on a ship headed for Saigon and Manila. In 1894, while he still worked for a French shipping company, he investigated an outbreak of plague in Hong Kong. After 7 days in a makeshift laboratory, he isolated the plague bacterium, which he called *Pasteurella pestis.*

Japanese bacteriologist Shibasaburo Kitasato had arrived in Hong Kong a few days before Yersin and also had isolated the bacterium. Kitasato published his findings in English and Japanese. Yersin published his in French. He also established a laboratory in Nha Trang, Vietnam, where he developed an antiplague serum that reduced the death rate from 90% to ≈7%. Since 1970, the organism has been called *Yersinia pestis*.

Sources: Burns W. Alexandre Yersin and his adventures in Vietnam. 2003; Medical Research Council National Institute for Medical Research. http://www.himr.mrc.ac.uk/millhillessays/2003/yersin/; http://www.whonamedit.com/doctor.cfm/2454.html; Dorland’s illustrated medical dictionary, 31st ed. Philadelphia: Saunders Elsevier; 2007.

